# Hydrogel-Based Hybrid Microcavity for a Plasmonic-Enhanced Laser Sensor

**DOI:** 10.1021/acssensors.5c03587

**Published:** 2025-12-22

**Authors:** Shuai Zhang, Matias Paatelainen, Arri Priimagi

**Affiliations:** Smart Photonic Materials, Faculty of Engineering and Natural Sciences, 7840 Tampere University, Tampere FI-33101, Finland

**Keywords:** hydrogel fiber, hybrid microcavity, lasing, localized surface plasmon resonance, humidity sensor

## Abstract

Hybrid microcavity systems combining optical resonators with responsive materials offer a promising route toward tunable, multifunctional photonic devices. Here, we demonstrate a hydrogel-based microfiber laser enhanced by plasmonic nanoparticles that enables single-mode operation with high sensitivity. The hydrogel network acts as a disordered scattering medium, inducing random lasing, while the microfiber geometry supports whispering-gallery modes with strong optical feedback. By tuning the microfiber diameter, we systematically investigate the interplay between microcavity modes and scattering. Incorporation of Au nanoparticles further enhances the optical confinement through localized surface plasmon resonances, providing single-mode lasing control. The resulting device exhibits strong humidity responsiveness and operational stability, with a sensitivity of 10^3^ pm/% RH and a rapid response time of 3.2 s. The results reported here provide a versatile approach for integrating smart materials and microcavities, advancing the development of ultrasensitive hydrogel-based photonic sensors.

## Graphical abstract

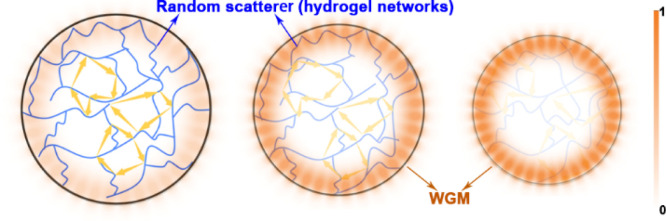

Hybrid microcavity resonators have recently emerged as powerful tools for photonic devices, enabling control of lasing characteristics through the integration of multiple distinct optical cavities.^1–3^ These systems promote enhanced light–matter interactions, multiwavelength operation, and mode-switchable lasing^4^ by exploiting synergistic or competitive coupling among various resonators, such as whispering gallery mode (WGM) resonators,^5,6^ random scattering structures,^7,9,8^ and distributed feedback microcavities.^10,11^ This design strategy effectively addresses limitations inherent to single-cavity systems, such as low emission efficiency^12^ and difficulty in single-mode selection,^13^ through the use of tunable geometries and materials with tailored optical properties.^14,15^ Hydrogels, characterized by three-dimensional porous networks and intrinsic scattering centers,^16,17^ serve as ideal candidates for scattering media,^18,19^ offering both tunability and biocompatibility,^20,21^ which are advantageous for tunable light sources and biochemical sensing applications.^8^ However, hybrid microcavity configurations that integrate hydrogel-based scattering media and resonators remain largely unexplored, particularly with respect to geometry-dependent coupling mechanisms that govern multimode interactions.

Noble metallic nanostructures, such as Au and Ag nanoparticles (NPs), exhibit exceptional optical properties due to their ability to support localized surface plasmon resonances (LSPRs).^22,23^ These resonances convert incident electromagnetic energy into surface plasmon polaritons, producing strong localized field enhancements at dielectric–metal interfaces when the excitation frequency matches the plasmon resonance.^24^ Such field confinement generates optical hotspots that significantly increase the figure of merit for trace-level detection.^25^ Furthermore, coupling between plasmonic modes and cavity resonances provides a novel mode selection mechanism, where collective in-phase oscillations of free electrons in the metal become coupled with resonator photons.^26,27^ These interactions have been exploited to realize high-throughput, ultrasensitive biochemical sensing platforms.^28,29^ Nevertheless, challenges remain regarding the long-term stability and sensitivity of smart-material-based plasmonic sensors, which currently limit their practical deployment.

In this work, we propose a hydrogel-based hybrid microcavity laser sensor that exploits the interplay between hydrogel scattering medium, WGM microcavities, and plasmonic enhancement to achieve high-sensitivity humidity detection. The intrinsic structural disorder within the hydrogel matrix functions as a random scattering medium, promoting random lasing via multiple feedback pathways. In our design, the microfiber structure functions as a WGM microcavity, providing high-quality-factor resonances that enhance photon confinement, foster competitive interactions among cavity modes, and enable polarization-dependent excitation. Combining Au NPs into Rhodamine 6G (R6G)-doped hydrogel precursor solution yields a coupled resonator–plasmonic system that enables single-mode selection. The fabricated device exhibited water-responsive properties intrinsic to the hydrogel, functioning as a humidity-sensitive laser sensor with a sensitivity exceeding 10^3^ pm/% RH and a response time of 3.2 s under humid conditions. This study presents a promising strategy for integrating smart materials, microcavities, and plasmonics into a unified platform, enabling the development of multifunctional, miniaturized optical sensors with high sensitivity and tunability for environmental monitoring and biochemical analysis.

## Results and Discussion

### Material and Fabrication

The poly­(*N*-isopropylacrylamide) (PNIPAm) hydrogel precursor was prepared by combining the hydrophilic monomer *N*-isopropylacrylamide (NIPAm) with the cross-linker *N*,*N*′-methylenebis­(acrylamide) (MBIS), using 2,2-dimethoxy-2-phenylacetophenone (DMPA) as the photoinitiator. Hydrogel-based microfibers were fabricated from this precursor solution, with R6G incorporated as the optical gain medium (Figure 1a). The PNIPAm microfibers were synthesized inside flexible silicone tubes with various diameters (300 μm, 600 μm, and 800 μm) using photoinitiated free-radical polymerization under uniform irradiation (365 nm, 1 mW cm^–2^ for 5 min). Optical micrographs and scanning electron microscopy (SEM) images confirmed the good uniformity of the R6G-doped PNIPAm microfibers, exemplified here by the 300-μm-diameter fiber (Figure 1b). The normalized absorption and photoluminescence spectra of R6G are shown in Figure 1c, and the pump source (532 nm) is denoted by a green line.

**Figure 1:**
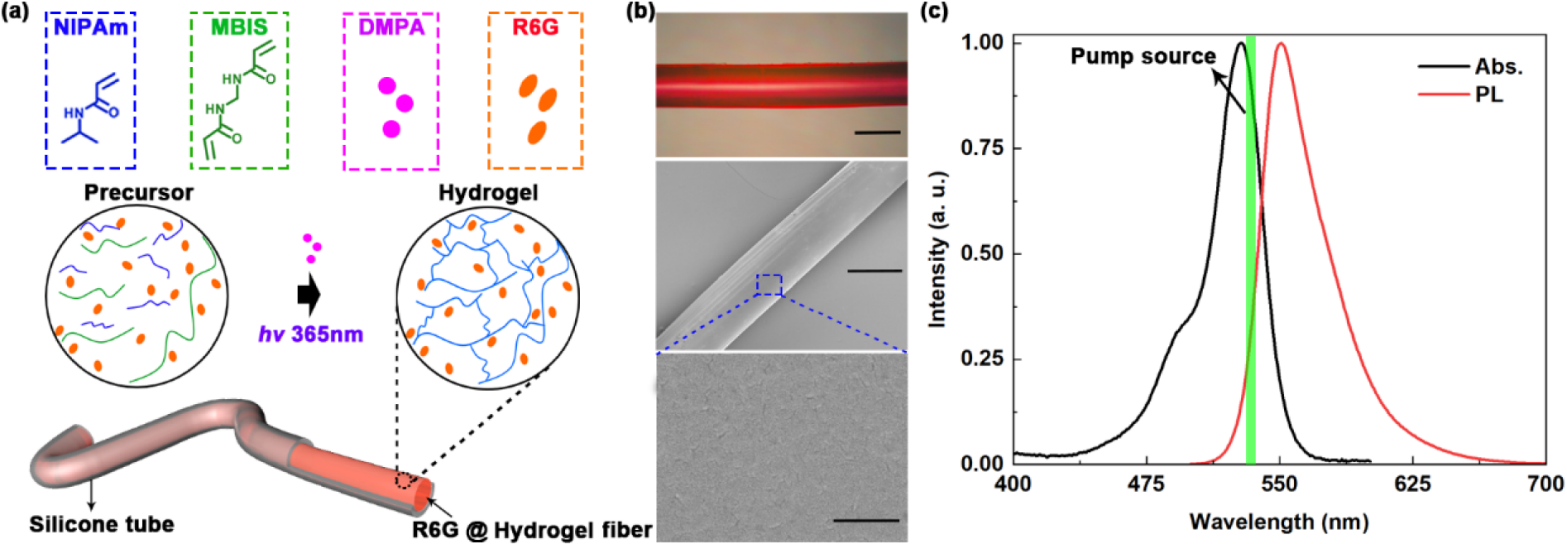
(a) Hydrogel fiber synthesis and fabrication scheme. (b) Optical micrograph (top) and SEM images (center and bottom) of an R6G-doped hydrogel microfiber with a 300- μm diameter. Scale bars: 300 μm (top and center) and 100 nm (bottom). (c) Normalized absorption spectrum (black) and photoluminescence (PL) spectrum (red) of R6G. The pump wavelength is shown by the green line.

### Investigation of Lasing Behavior in Hybrid Microcavity

As schematically illustrated in Figure 2a, the blue lines represent hydrogel networks formed by photopolymerization, while the WGM originates from the microfiber cavity. Upon optical excitation, the dye-doped hydrogel microfiber emitted fluorescence with the hydrogel network providing random scattering centers to form closed-loop random feedback. Simultaneously, the WGM microcavity offered strong optical confinement via continuous total internal reflection at the smooth, curved inner boundary of the microfiber, thereby amplifying the lasing output. For comparison, the emission of the flat hydrogel film reveals incoherent random lasing behavior without feedback enhancement from the microcavity (Figure S1). Notably, resonance enhancement from the WGM microcavity occurs only when the internal random lasing modes are both frequency- and spatially matched with the resonance condition of the microcavity. In this sense, the hydrogel-based microcavity behaves as a mode-selective resonator, where localized feedback from internal scattering enhances only the modes that satisfy the resonance condition. The spatial variation of the spectra reflects the random nature of the light confinement, while the temporal stability indicates the structural robustness of the scattering network. As the resonator volume decreases, the effective mode volume is reduced, leading to an increased optical field density and confinement efficiency through Purcell-effect enhancement, as discussed later.

**Figure 2:**
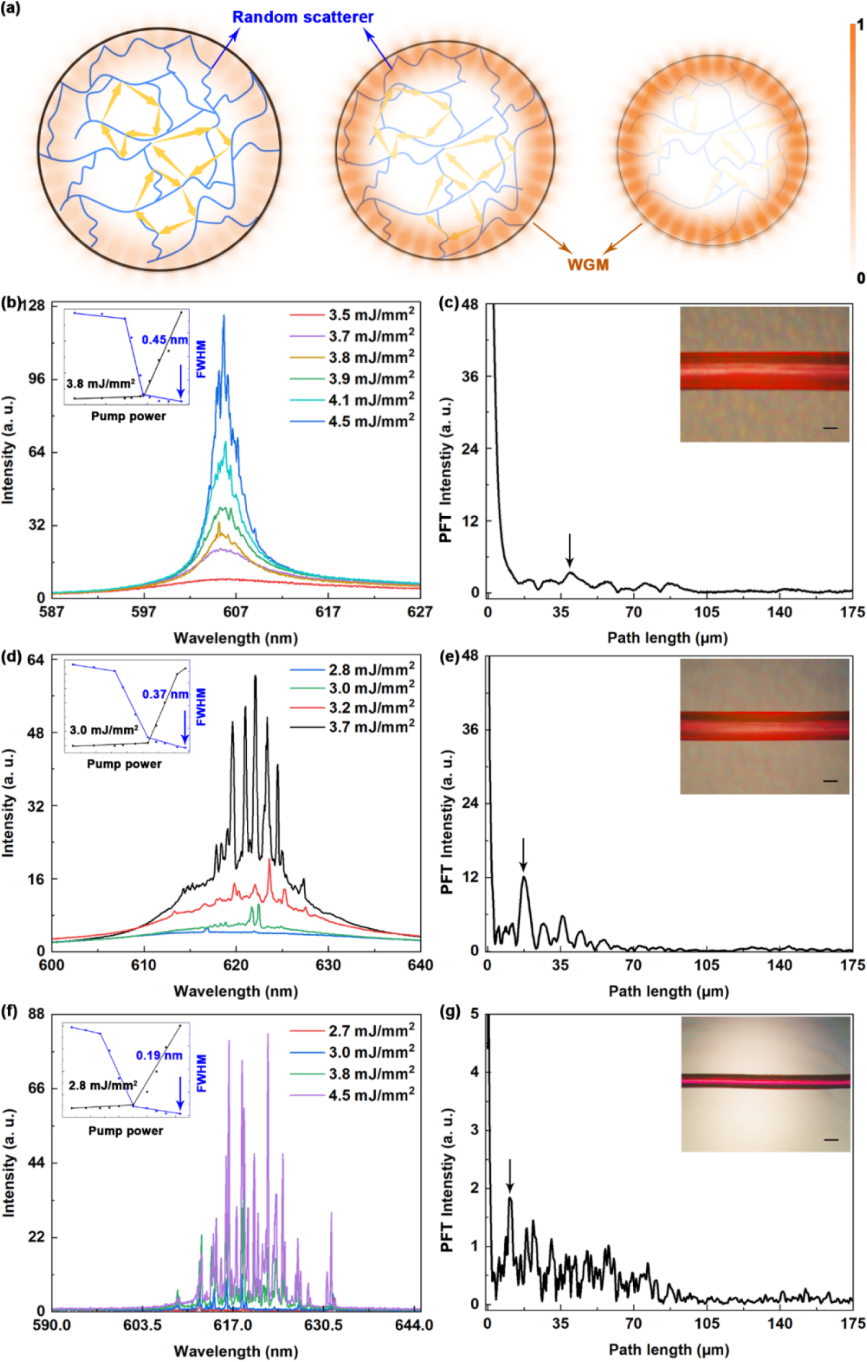
(a) Schematic illustration of the interaction between the WGM microcavity and hydrogel network scattering. (b), (d), and (f) Emission spectra of hydrogel fibers (800 μm, 600 μm, 300 μm) under different pump energy densities. Insets: Emission intensity and line width of the random lasing as a function of pump energy, with red lines denoting lasing thresholds. (c), (e), and (g) Power Fourier Transform (PFT) of the random lasing spectra at 4.1 mJ cm^–2^. Insets: Optical micrographs of hydrogel fiber random lasers with different diameters (800 μm, 600 μm, 300 μm). Scale bar: 300 μm.

As the pump energy density increased, the emission spectra transitioned from fluorescence to random lasing, as shown in Figure 2b, 2d, and 2f. Distinct variations in emission characteristics were observed among hydrogel microfiber random lasers of different diameters (800 μm, 600 μm, 300 μm). A pronounced reduction in both lasing line width (0.45 nm, 0.37 nm, 0.19 nm) and threshold (from 3.8 mJ mm^–2^, 3.0 mJ mm^–2^, 2.8 mJ mm^–2^) was observed, with the corresponding threshold behavior and line width narrowing as shown in the insets. Analysis of the emission spectra further reveals that decreasing the microfiber cavity diameter leads to a transition from sparse to dense laser modes, accompanied by a marked enhancement in resonant feedback within the hybrid microcavity system.

The power Fourier Transform (PFT) provides effective means for analyzing the equivalent cavity of random lasers by indicating the strength of light scattering.^30^ The abscissas corresponding to the peaks of the PFT spectra represent the Fourier components from which the cavity length can be estimated using the expression *L* = π*d*_*m*_/*mn*_eff_, where *n*_eff_ is the effective refractive index of the sample, and *m* is the order of the Fourier harmonic. For the present analysis, the fundamental Fourier component (*m* = 1) was used to calculate the optical path length. Figure 2c, 2e, and 2g show the PFT spectra of hydrogel fiber random lasers (excited at 4.1 mJ mm^–2^) with diameters of 800 μm, 600 μm, and 300 μm, respectively. In Figure 2c, a weak peak appears at around 39 μm, indicating a low feedback coherence strength. Notably, stronger confinement within smaller microfibers significantly enhanced the feedback coherence strength, as evidenced by the pronounced scattering shown in Figure 2e and 2g. As the microfiber diameter decreased to 600 μm and 300 μm, the optical path length shortened to 17 μm and 10 μm, respectively, clearly presenting improved scattering behavior. The insets illustrate the good optical uniformity of the dye-doped hydrogel fibers. These results demonstrate that the geometric parameters of the microfiber play a determining role in governing the emission properties of the hydrogel-based random lasers.

To further investigate the role of the microfiber cavity, we examined the emission characteristics of the hydrogel microfiber under different pump polarizations (Figure 3). Figure 3a and 3b present the lasing spectra of the hydrogel microfiber with a diameter of 300 μm for pump polarizations parallel and perpendicular to the fiber axis, with corresponding lasing thresholds of 3.6 mJ mm^–2^ and 4.0 mJ mm^–2^ (Figure 3c). Figure 3d shows the polarization-dependent lasing intensities for the hydrogel microfibers with varying diameters. The dependence of the emission intensity on the pump polarization originates from variations in coupling efficiency between the pump field and the cavity modes. When the pump polarization is parallel to the fiber axis, its electric field aligns with the dominant optical modes, resulting in more efficient excitation of the gain medium and consequently stronger lasing emission. As the fiber diameter decreases, the enhanced optical confinement and field localization amplify the influence of the cavity’s intrinsic anisotropic feedback, leading to the emission polarization being predominantly governed by the cavity.

**Figure 3:**
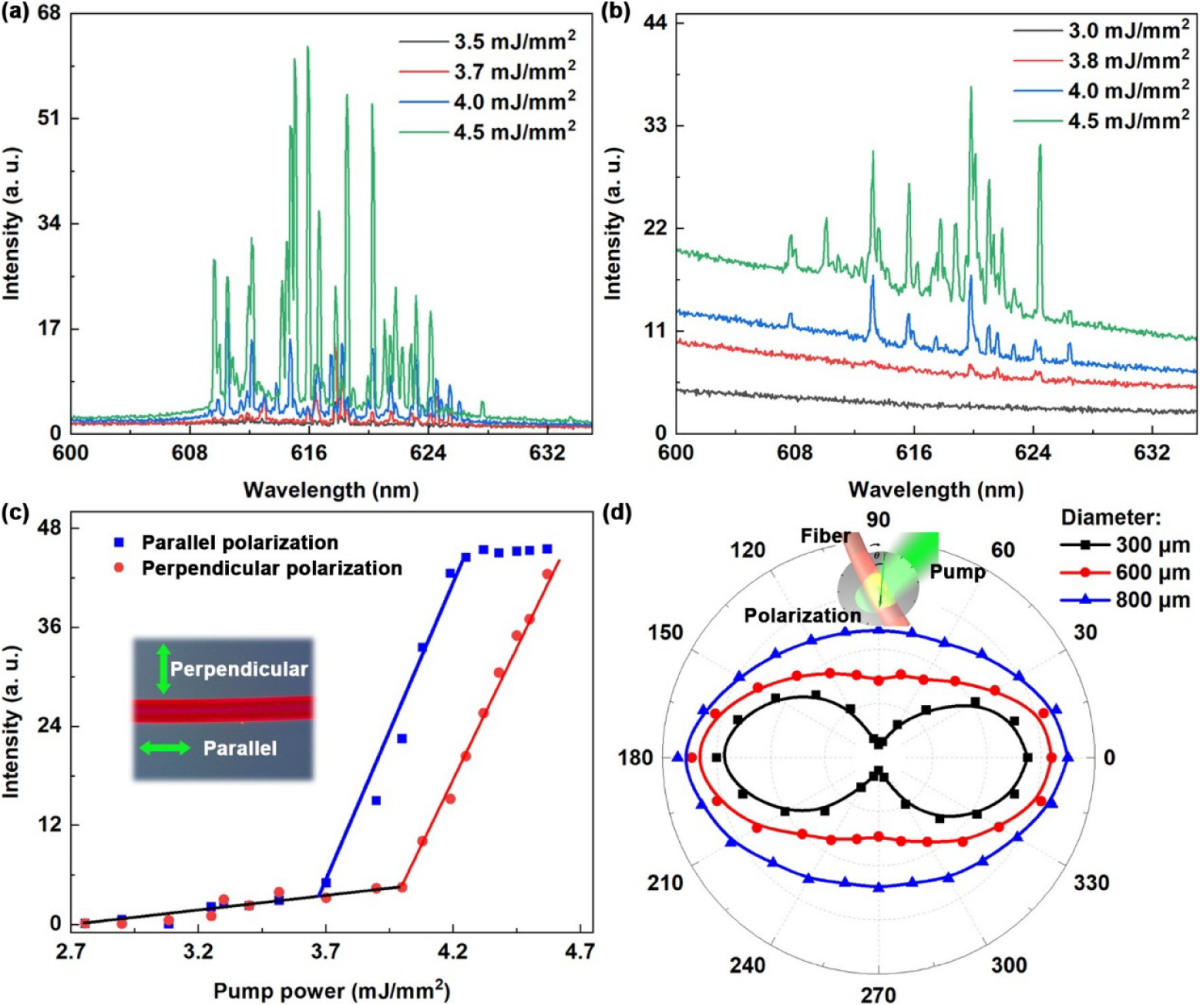
(a,b) Emission spectra of the hydrogel microfiber under different pump energy densities for parallel (a) and perpendicular (b) pump polarization. (c) Emission intensity and line width of the random lasing as a function of pump fluence with excitation using parallel and perpendicular polarization with respect to the fiber axis highlighting the emission efficiency difference under different conditions, as depicted in the inset. The green arrows indicate the direction of pump polarization. (d) Polarization-dependent lasing intensity characteristics of the hydrogel fiber laser. Insets: The relative angle between the pump polarization and the axial direction of the sample.

The results demonstrate that the WGM microcavity amplifies randomized scattering processes within the hydrogel matrix. This enhancement of feedback coherence, combined with improved excitation efficiency in the gain medium, results in elevated emission intensity and spectral coherence through Purcell effect-mediated photon confinement.^31^ In our case, the use of a microfiber cavity with a smaller diameter enhanced the Purcell factor by reducing the optical mode volume, thereby strengthening the photon confinement. Additionally, parallel-polarized excitation light improved the spatial overlap with the cavity modes, resulting in increased excitation efficiency.

### Lasing Properties Controlled by Doping with Au NPs

Mode selection plays a crucial role in integrated photonic systems, where stable and tunable optical resonant mode operation is highly desirable for on-chip light sources and sensing. Controlling the lasing mode through metal nanoparticles therefore offers a promising route toward functional and highly sensitive photonic devices. Plasmonic-enhanced hydrogel microfibers with a diameter of 300 μm were fabricated by incorporating varying volumes of 50 nm Au NP dispersion into the hydrogel precursor (Table 1, Figure S2). As shown in Figure 4, the emission properties, including the lasing mode number and thresholds, can be effectively controlled by the Au NP concentration. Because Au NPs have an absorption cross-section larger than the scattering cross-section, the *Q*-factor of the resonator decreases compared to that of pristine microfiber, primarily due to the additional scattering losses. Therefore, single-mode lasing arises from hybrid resonant modes once most competing modes are dissipated.

**Figure 4:**
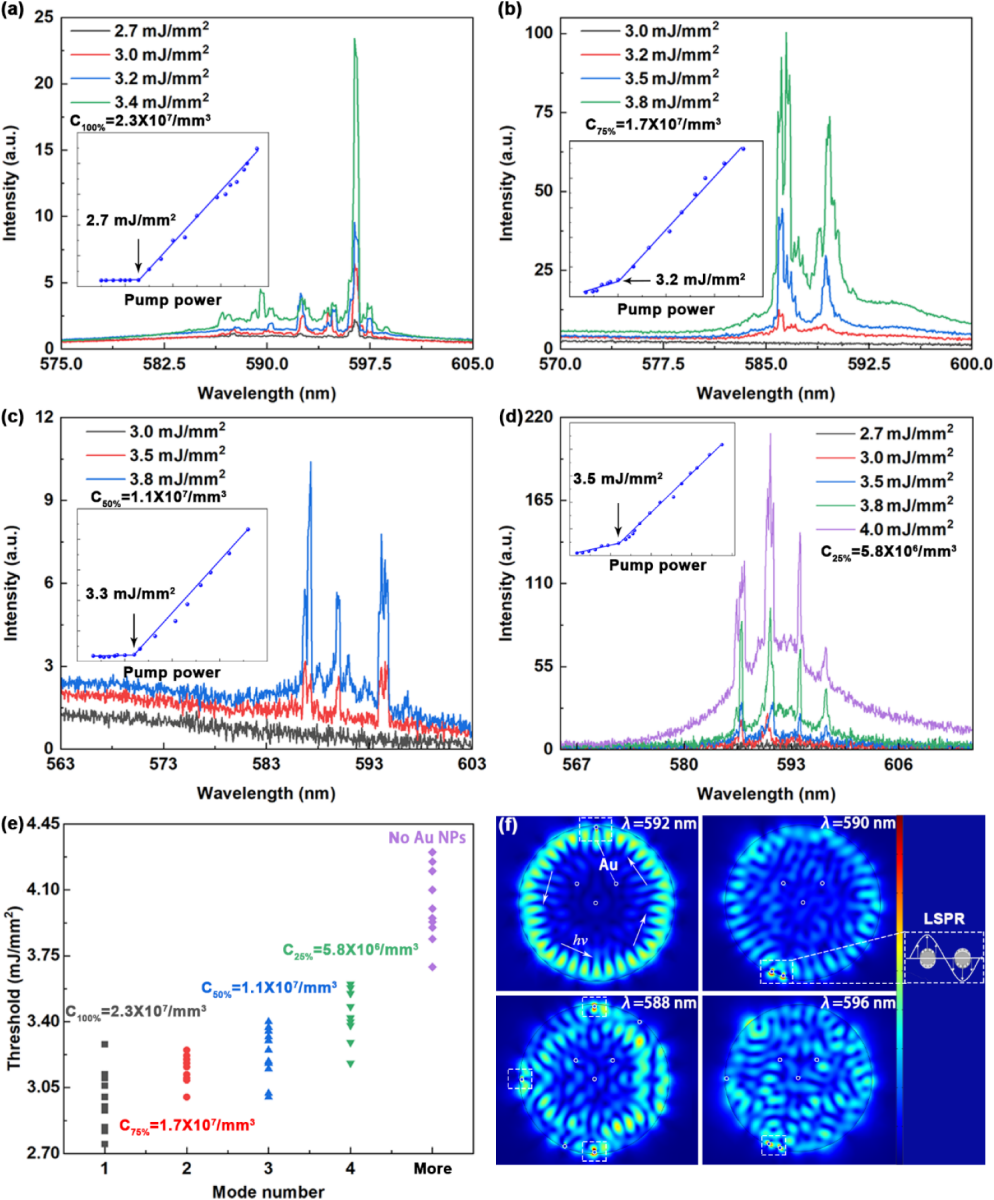
(a–d) Emission spectra of plasmonic-enhanced hydrogel fiber random lasers at distinct Au NP concentrations: ((a) *C*_100%_ = 2.3 × 10^7^ mm^–3^, (b) *C*_75%_ = 1.7 × 10^7^ mm^–3^, (c) *C*_50%_ = 1.1 × 10^7^ mm^–3^, and (d) *C*_25%_ = 5.8 × 10^6^ mm^–3^) under different pump energy densities. Insets: The corresponding lasing thresholds. (e) Lasing thresholds of plasmonic-enhanced hydrogel fiber lasers with varying emission mode numbers and Au NP density in different microfibers. (f) Simulation of the plasmonic-enhanced fiber cavity with varying Au NP concentrations, along with a schematic illustration of LSPRs excited in the Au NPs.

**Table 1:** Volume Proportion of H_2_O and Au NP Dispersion

Material proportion	H_2_O (v/v)	Au NP dispersion (v/v)	Au NP density in microfiber (/mm^3^)
Initial hydrogel fiber	100%	0	0
4-Mode hydrogel fiber	75%	25%	5.8 × 10^6^
Trimode hydrogel fiber	50%	50%	1.1 × 10^7^
Dual-mode hydrogel fiber	25%	75%	1.7 × 10^7^
Single-mode hydrogel fiber	0	100%	2.3 × 10^7^

Doping the microfiber with Au NPs at different concentrations introduces tunable scattering losses, enabling control over the number of lasing modes. As shown in Figure 4a–4d, single-mode, dual-mode, tri-mode, and four-mode lasing were observed, with thresholds of 2.75 mJ cm^–2^, 3.25 mJ cm^–2^, 3.35 mJ cm^–2^, and 3.51 mJ cm^–2^, respectively. The single-mode lasing exhibited the lowest threshold, which can be attributed to the LSPR mechanism. When the LSPR frequency of the Au NPs at the hydrogel microfiber surface coincides with the internal cavity resonance modes, the LSPR effect enhances the local optical field, thereby lowering the lasing threshold (Figure S3). Samples with different Au NP concentrations (C_100%_, C_75%_, C_50%_, and C_25%_) and the undoped control displayed distinct emission characteristics. Based on these differences, the samples were grouped into four categories, and their corresponding lasing thresholds are summarized in Figure 4e. The single-mode hydrogel fibers exhibited the lowest average threshold, making them particularly suitable for the sensing experiments described in the following section. The finite element method was employed using COMSOL to investigate the distortion of resonant mode distributions in the microcavity induced by LSPR, as well as the corresponding local ***E***-field enhancement (Figure 4f). In the simulations, the Au NPs were randomly distributed around the microfiber cavity to emulate the experimental condition. The results presented that the increased number of additional Au NPs near the microfiber cavity led to the increased distortion of the resonant mode distribution, resulting in resonant mode selection, while the LSPR effect exhibited ***E***-field enhancement at the matched frequency. The comparative results (Figure S4) confirm that the scattering of Au NPs governed the optical mode selection, while their LSPR effect simultaneously enhanced the optical gain, leading to a synergistic improvement in the lasing performance.

### Humidity Sensing with Hydrogel Microfiber Lasers

The humidity response of the plasmonic-enhanced hydrogel microfiber laser was evaluated by using a humidity-controlled chamber. Figure 5a shows a schematic illustration of the humidity sensing mechanism. The presence of Au NPs on the fiber surface induces LSPR enhancement, thereby enabling the formation of a single-mode hydrogel microfiber sensor with high sensitivity. The humidity-dependent response of the plasmonic-enhanced microfiber sensor is shown in Figure 5b. The sensor was first stabilized in an environment with a constant humidity of 26% RH and then rapidly transferred to a chamber with 40% RH. As a result, the resonant wavelength shifted from 604.5 nm to 589.6 nm. As shown in Figure 5c, the response time of the plasmonic-enhanced microfiber sensor was estimated to be 3.5 s for a humidity change of 14% RH.

**Figure 5:**
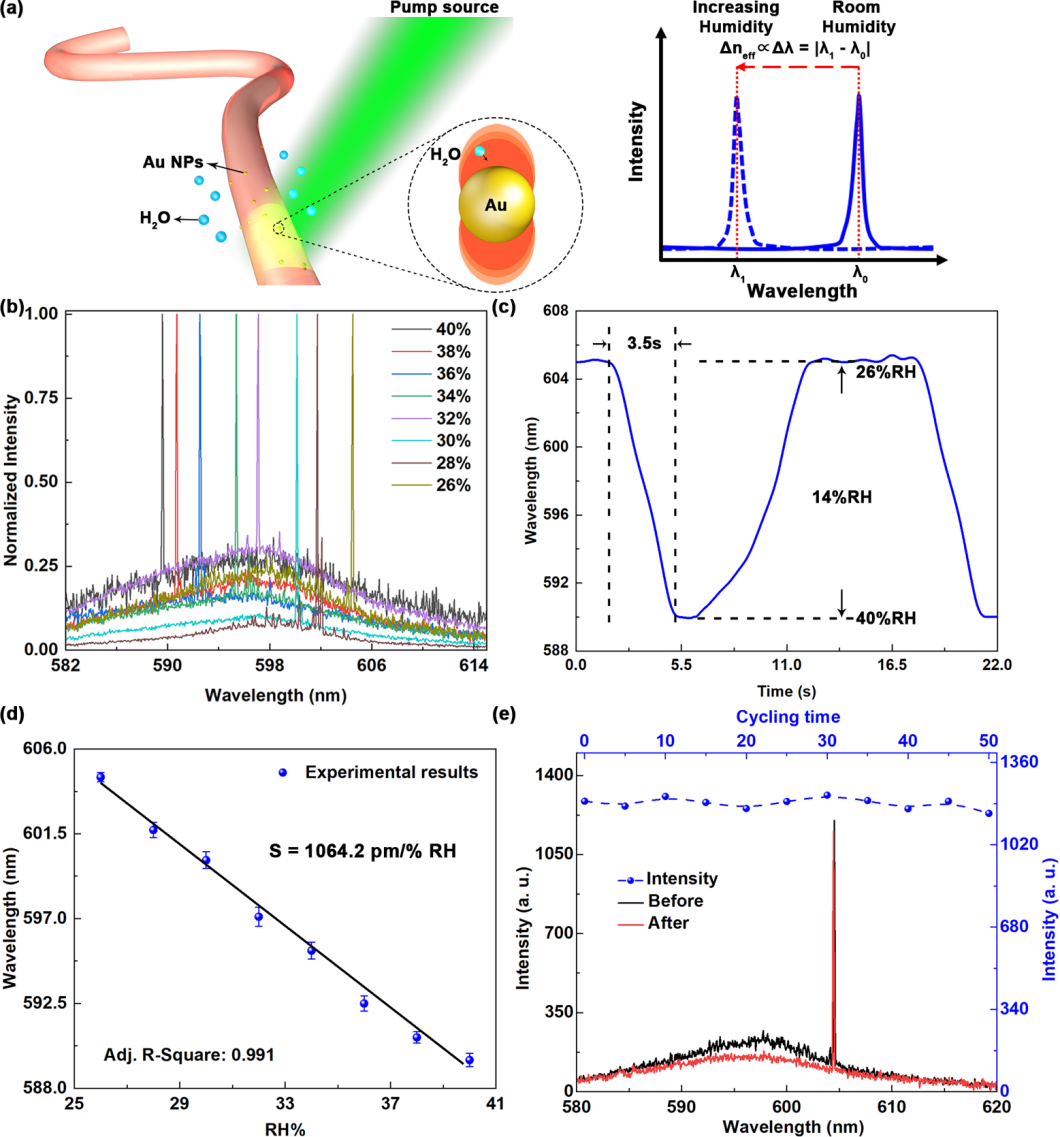
(a) Scheme and principle of the plasmonic-enhanced microfiber in a humidity sensing experiment. (b) Wavelength shift in the single-mode hydrogel microfiber with increasing humidity. The microfiber diameter is approximately 300 μm. (c) Emission wavelength tuning range of the single-mode hydrogel microfiber. (d) Time-dependent response of the single-mode microfiber sensor. (e) Lasing spectra of the single-mode hydrogel microfiber before and after multiple cycling experiments and emission intensity properties recorded in the cycling test.

To study the sensing stability of the proposed plasmonic-enhanced microfiber sensor, the devices were tested in different chambers with varying humidity levels. A pronounced resonant peak blueshift from 605 nm to 590 nm was observed when the humidity increased from 26% to 40%. The corresponding sensitivity was measured as 1064.2 pm/% RH (Figure 5d). The effective refractive index *n*_eff_ of the device decreased with increasing RH, which can be ascribed to the water absorption of the hydrogel.^32^ The resulting shift in resonant mode can be expressed as:^33^

1 $$\frac{\Delta \lambda }{\lambda }=\frac{\Delta n}{n_{\mathrm{eff}}}+\frac{\Delta D}{D}$$

where Δλ is the wavelength shift, and Δ*n* and Δ*D* are the variations in the refractive index and the diameter of the microfiber laser, respectively. In this process, the variation of $\frac{\Delta n}{n_{\mathrm{eff}}}$ played a major role in determining the blueshift in the resonant wavelength (Figure S5). These observations are consistent with prior reports^34,35^ showing that hydrogels swell and reduce their refractive index upon water uptake, thereby shifting cavity resonances, while Au NPs’ LSPR peaks shift with changes in the local refractive index—effective refractive index n_eff_ change is the primary factor for the observed spectral blueshift.^36^

Because of the plasmonic enhancement, the microfiber sensor showed a dramatic blueshift with increasing RH. The shift in the resonant wavelength is given by^37^

2 $$\frac{\Delta \lambda }{\lambda }≅-\frac{\varepsilon {|E|}^{2}}{2V{|E_{0}|}^{2}}$$

where ε is the cavity permittivity, *E* is the WGM electric field strength, and *V* is the optical mode volume of the resonator.

As shown by eq 2, the resonance wavelength shift increases proportionally to the local electric field intensity |*E*|^2^. Acting as “hot spots” in the evanescent field, the additional Au nanospheres on the microfiber formed an LSPR-coupled system that enhanced the local field. The resonant wavelength shift increased remarkably compared with the initial microfiber (Figure S6), leading to a dramatic improvement in detection sensitivity. A comparison of the emission characteristics is presented in Figure 5e. Importantly, the optical properties of the proposed device did not change significantly; namely, the resonant wavelength and emission intensity remained essentially unchanged after dozens of cycle testing experiments. These results confirm that the device exhibits excellent sensing stability.

The limit of detection (LOD) is an important standard for sensors.^38^ The LOD can be calculated using

3 $$\mathrm{LOD}=3\times \frac{\sigma }{S}$$

where *S* is the sensitivity of the sensor, and the standard deviation of the regression line is given by $\sigma =\sqrt{\frac{1}{N}\overset{N}{\underset{1}{\sum }}{\left(x_{i}-\mu \right)}^{2}}$ ; μ is the average value. The corresponding sensitivity was measured as 1064.2 pm/% RH. The LOD of the proposed plasmonic-enhanced sensor is 0.05% RH. During this humidity interval, the emission wavelength can be distinguished clearly.

## Conclusions

In summary, we demonstrated a novel laser sensor by integrating plasmonic nanoparticles into a microcavity system. In this configuration, the hydrogel network acted as random scattering media, coupling the random scattering with WGM microcavities. Combining plasmonic enhancement provided by the Au nanoparticles and controlled mode selection, we realized a humidity-responsive microlaser with high sensitivity (10^3^ pm/% RH) and fast response time (3.2 s). These results underscore the potential of this approach for real-time environmental monitoring. Beyond humidity sensing, the dual role of the hydrogel, acting as both a functional element and a scattering medium, suggests that this strategy can be generalized to other stimuli-responsive systems. By employing materials that respond to temperature, pH, or light, similar hybrid microcavity platform could be developed for adaptive and tunable photonics. More broadly, our work highlights how combining smart materials and tailored microcavity architectures can enable compact, multifunctional photonic devices, paving the way for next-generation sensing platforms.

## Experimental Section

### Hydrogel Fiber Fabrication

The hydrogel fibers were prepared via free-radical photopolymerization from a precursor solution inside a silicone tube. First, the precursor solution was prepared by dissolving NIPAm (95.5 mol %), MBIS (4 mol %), DMPA (Irgacure 651) (0.5 mol %), and R6G (Sigma-Aldrich) (1.8 mg/mL) in a 2:1 1,4-dioxane–water mixture with a total mass concentration of 500 mg/mL. To ensure thorough mixing, the solution was subsequently sonicated for 10 min at ambient temperature and purged with nitrogen for 10 min before use to remove dissolved oxygen.

The polymerization was carried out at ambient temperature by capillary filling of the silicone tube and illuminating from the top with a 365  nm LED (CoolLED pE-4000 16 LED) at 1 mW/cm^2^ intensity for 5 min under N_2_ flow. After polymerization, the silicone tube was opened with a razor. Finally, the samples were dried under vacuum at 40 °C for 12 h to remove excess water inside.

### Plasmonic-Enhanced Microfiber Fabrication

The fabrication is the same as that of the hydrogel fiber fabrication, including the precursor solution preparation and polymerization. The 50-nm Au NP dispersion (Nanopartz, 30-10-750) was mixed with the hydrogel precursor solution mentioned above. The volume proportion of H_2_O and Au NPs dispersion is shown in Table 1. Then, the mixed solution was subsequently sonicated for 10 min at ambient temperature and purged with nitrogen for 10 min before use to remove dissolved oxygen. The polymerization was the same as that of the hydrogel fiber fabrication.

### Humidity Sensing Setup

The setup consists of a transparent chamber (6.5 × 6.5 × 10 cm) equipped with a wall-mounted minisize digital humidity detector (Herbgarden). A silicone tube with an inner diameter of approximately 1 cm is used to deliver moisture from an external humidifier (Beurer, LA40) into the chamber. During humidity sensing experiments, the entire system is sealed using a transparent plastic film of uniform thickness to maintain a relatively stable internal humidity level.

### Optical Characterization

Optical pumping of the hydrogel microfibers and lasers was done with frequency tripled Nd:YAG laser (Quanta-Ray Lab-130-10, Spectra-Physics, repetition rate of 10 Hz) operating with an optical parametric oscillator (versaScan/MB, Spectra-Physics) that converted the output to 532 nm and 5 ns pulse. Lasing was studied on an inverted microscope (Zeiss) by placing the sample on a glass slide. A pump beam with a diameter exceeding 800 μm was focused onto the sample from below using a 20× microscope objective, ensuring full coverage of the sample. The emission signal was collected through the same objective and a beam splitter into a spectrometer (SR-303i-B/Newton CCD, Andor). All experiments were conducted at room temperature.

### Calculation and Simulation

The PFT calculation is executed in the Origin software (Origin 2023b 64-Bit). The optical mode simulation is conducted by COMSOL software. The Au material model is based on Johnson and Christy (1972). A dielectric material with a constant refractive index *n*_c_ = 1.55 was set as the inside medium of the microfiber (hydrogel). The simulation was conducted under ambient conditions (*n*_s_ = 1).

## Supplementary Material



## Notes

Random lasing in hydrogel films, density of Au NPs, extinction spectra of Au nanoparticles, emission spectra comparison, relationship between the microfiber diameter and humidity, humidity response of the initial hydrogel microfiber (PDF)
