# Supplementary material for: Hydrogel-Based Hybrid Microcavity for a Plasmonic-Enhanced Laser Sensor
Source: ACS Sens. 2025 Dec 22;11(1):644–52. doi: 10.1021/acssensors.5c03587 (PMC12836339; doi:10.1021/acssensors.5c03587)
Supplement: Supplementary file 1 [file se5c03587_si_001.pdf]

Supporting Information

Hydrogel-based Hybrid Microcavity for Plasmonic-enhanced Laser Sensor

Shuai Zhang, Matias Paatelainen, Arri Priimagi*

Corresponding Author:

Arri Priimagi - Smart Photonic Materials, Faculty of Engineering and Natural Sciences, Tampere University, Tampere FI-33101, Finland.

Email: arri.priimagi@tuni.fi

Authors:

Shuai Zhang - Smart Photonic Materials, Faculty of Engineering and Natural Sciences, Tampere University, Tampere FI-33101, Finland.

Matias Paatelainen - Smart Photonic Materials, Faculty of Engineering and Natural Sciences, Tampere University, Tampere FI-33101, Finland.

CONTENTS

1. Random lasing in hydrogel films

2. Density of Au nanoparticles

3. Extinction spectra of Au nanoparticles

4. Emission spectra comparison

5. Relationship between the microfiber diameter and humidity

6. Humidity response of the initial hydrogel microfiber

1. Random lasing in hydrogel films

The lasing behavior of flat hydrogel films is presented in Figure S1. The flat hydrogel film was fabricated by filling hydrogel precursor solution into the glass chamber with a thickness of 20 μm.

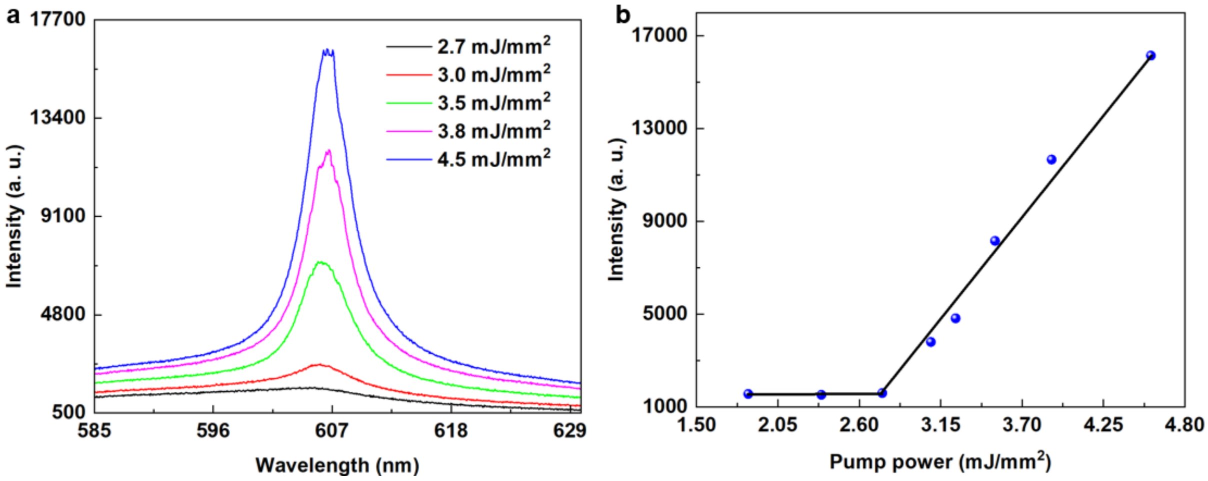

Figure S1 (a) Emission spectra of hydrogel film. (b) Emission intensity of the random lasing as a function of the pumping fluence.

2. Density of Au nanoparticles

The silicon sheets (1 cm × 1 cm × 0.05 cm) were employed as conductive substrates.

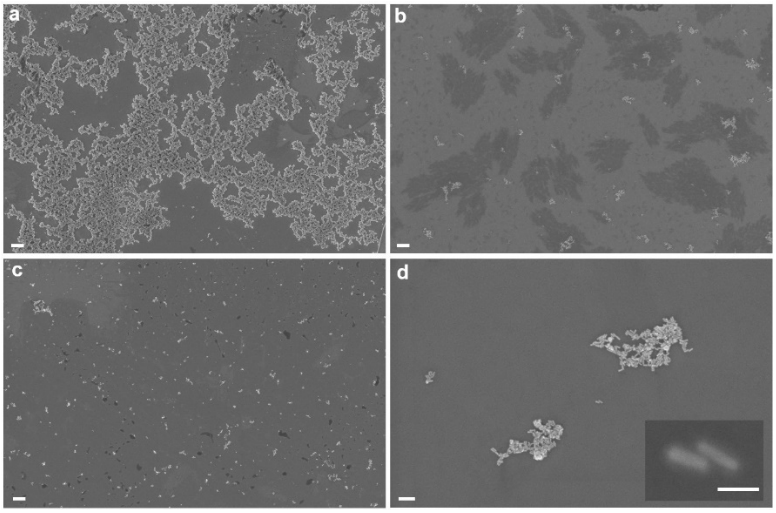

Figure S2 (a) SEM image of original Au NPs dispersion without dilution. The scale bar is 2 μm (b) and (c) SEM image of Au NPs per unit volume after 2:1 and 3:1 dilution of deionized water to nanoparticle dispersion. The scale bar is 2 μm (d) Enlarged SEM image of Au NP clusters and the scale bar is 200 nm. The scale bar of inset is 50 nm.

The density of Au nanoparticles (NPs) is calculated by:

$$C = \frac{N}{V}$$

C is the density of Au NPs in per unit of volume and N is number of Au NPs in V.

The Au NP density in plasmonic-enhanced microfiber is $C_{25\%} \approx 5.8 \times 10^6/\mathrm{mm}^3$, $C_{50\%} \approx 1.1 \times 10^7/\mathrm{mm}^3$, $C_{75\%} \approx 1.7 \times 10^7/\mathrm{mm}^3$, $C_{100\%} \approx 2.3 \times 10^7/\mathrm{mm}^3$, respectively, as shown in **Table1**.

3. Extinction spectra of Au NPs

For absorption measurements, Au NP dispersion droplets were applied directly onto a clean glass substrate, and the sample was placed on hotplate at 150°C for drying. To ensure adequate signal intensity, the dispersion was used without dilution during sample preparation. The absorption spectra of the Au NPs samples were measured in ambient conditions (Cary 60 UV–vis, Agilent Technologies).

Figure S2 shows that the absorbance maximum occurs near 720 nm, attributed to the red shift induced by the aggregation of Au NPs. The emission center of plasmonic-enhanced hydrogel microfiber is located around 600 nm, corresponding to the spectral region where the LSPR of the Au nanoparticles exhibits strong field enhancement.

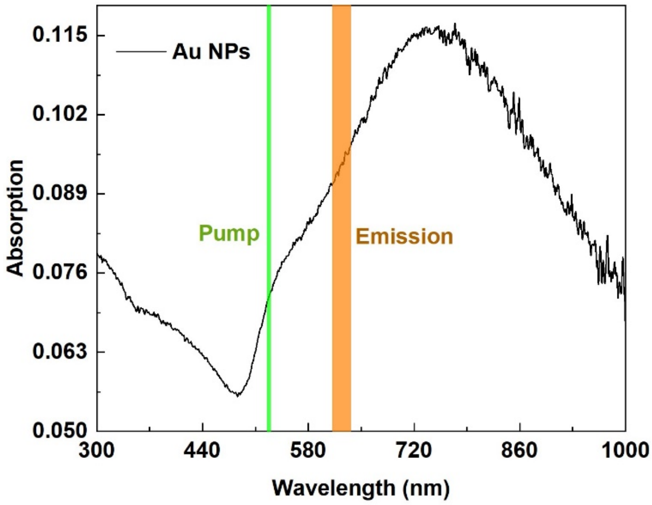

Figure S3 The absorption of Au NPs (particle clustering) deposited on glass substrates.

4. Emission spectra comparison

For comparison, the lasing spectrum of dielectric nanoparticles (TiO_2, 50 nm, Sigma-Aldrich) when coupled to a microfiber is illustrated by the red line in Figure S4. Under the same pumping conditions (3.2 mJ mm^{-2}), the emission intensity of the plasmonic-enhanced microfiber was enhanced significantly by the LSPR and was more than 2 times higher than that of the microfiber with TiO_2 NPs.

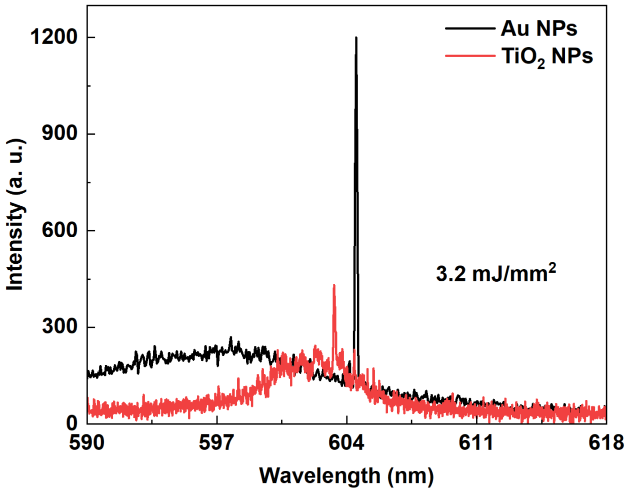

Figure S4 Comparison between the emission spectra of the plasmonic-enhanced microfiber and the TiO_2-equipped microfiber. Black: Au NPs equipped sample. Red: TiO_2 NPs equipped sample.

5. Relationship between the microfiber diameter and humidity

The relationship between the microfiber diameter and humidity was explored in the homemade humidity chamber by using microscope (20x, Zeiss). The diameter increases by 0.6 μm as the relative humidity rises from 26% to 60%.

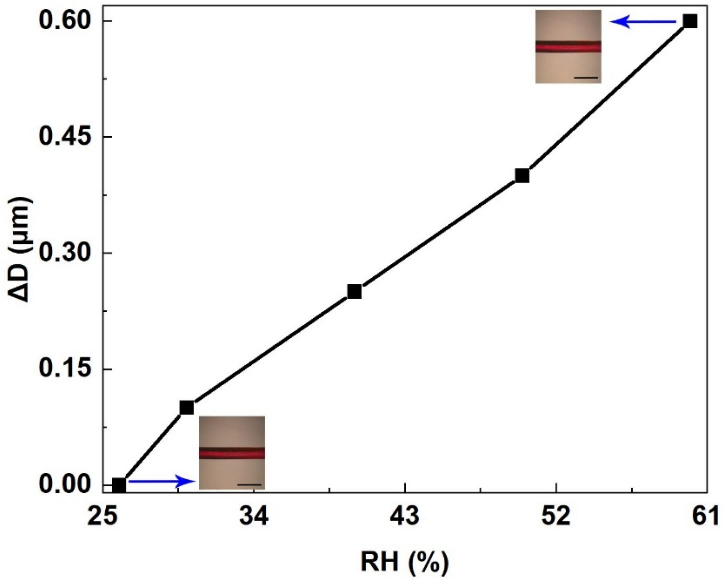

Figure S5 Relationship between the microfiber diameter and relative humidity. Scale bar: 600 μm.

6. Humidity response of the initial hydrogel microfiber

The humidity response of the microfiber sensor was analyzed (Figure S3). The lasing peak shifted from 597 nm to 601 nm when the humidity varied from 26% to 41%. The sensitivity of the initial hydrogel microfiber sensor is 266.7 pm/%RH which is lower than that of plasmonic-enhanced microfiber sensor.

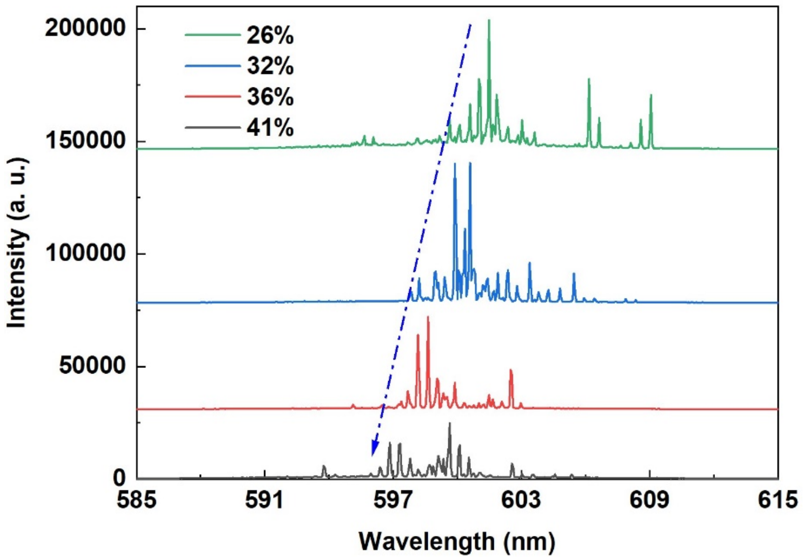

Figure S6 The humidity response of the microfiber sensor without Au NPs.
